# Stages of Change Profiles among Adults Experiencing Hearing Difficulties Who Have Not Taken Any Action: A Cross-Sectional Study

**DOI:** 10.1371/journal.pone.0129107

**Published:** 2015-06-04

**Authors:** Vinaya Manchaiah, Jerker Rönnberg, Gerhard Andersson, Thomas Lunner

**Affiliations:** 1 Department of Speech and Hearing Sciences, Lamar University, Beaumont, Texas, United States of America; 2 Linnaeus Centre HEAD, Swedish Institute for Disability Research, Department of Behavioural Sciences and Learning, Linköping University, Linköping, Sweden; 3 Audiology India, Mysore, Karnataka, India; 4 Department of Clinical Neuroscience, Division of Psychiatry, Karolinska Institutet, Stockholm, Sweden; 5 Eriksholm Research Centre, Oticon A/S, 20 Rørtangvej, Snekkersten, Denmark; Sun Yat-sen University, CHINA

## Abstract

The aim of the current study was to test the hypothesis that adults experiencing hearing difficulties who are aware of their difficulties but have not taken any action would fall under *contemplation* and *preparation* stages based on the transtheoretical stages-of-change model. The study employed a cross-sectional design. The study was conducted in United Kingdom and 90 participants completed University of Rhode Island Change Assessment (URICA) scale as well as measures of self-reported hearing disability, self-reported anxiety and depression, self-reported hearing disability acceptance, and provided additional demographic details online. As predicted, the results indicate that a high percentage of participants (over 90%) were in the contemplation and preparation stages. No statistically significant differences were observed among groups of stage with highest URICA scores and factors such as: years since hearing disability, self-reported hearing disability, self-reported anxiety and depression, and self-reported hearing disability acceptance. Cluster analysis identified three stages-of-change clusters, which were named as: *decision making* (53% of sample), *participation* (28% of sample), and *disinterest* (19% of sample). Study results support the stages-of-change model. In addition, implications of the current study and areas for future research are discussed.

## Introduction

Health behaviour change refers to facilitating changes of habits and/or behaviour related to health. There are several models proposed which provide a theoretical framework when studying and understanding health behaviour change [1, 2], and one example is the Transtheoretical Model of Change (TTM) [3–6]. Though some researchers suggest that the health behaviour change models could be useful in audiology research and practice [7–11], few empirical studies exist [12–18]. These studies have generally used either the Health Belief Model (HBM) and/or TTM to study beliefs of college students in relation to exposure to loud music [14], attitudes of medical practitioners towards hearing rehabilitation of older adults [15], readiness and attitudes of tinnitus patients to change their behaviour [16], and attitudes to hearing help-seeking and stages of change through this process [12, 13, 17, 18].

### Transtheoretical Model of Change

The transtheoretical (also known as stages-of-change) model is based on the assumption that behaviour change is achieved via various stages and it mainly focuses on an individual’s readiness to make a change [19]. The model was originally developed by Prochaska and DiClemente when they were studying how smokers were able to give up their smoking habits or addiction [3]. The transtheoretical concepts include various aspects such as process of change, decision imbalance, self-efficacy, and temptation [20]. However, we have focused specifically on the stages of change aspect of this model in the current study. Over the years different versions of this model have been proposed. However, a four-stage model has been used most often to describe different stages of change [21]. The four stages include: (1) *precontemplation*—not thinking seriously about changing a specific behaviour and not interested in help (i.e., often in denial); (2) *contemplation*—aware of the consequences of the problem and spends time thinking about the problem; (3) *action*—taking active steps to change their behaviour; and (4) *maintenance*—successfully avoiding any temptation to give up the change they have made. In some stages-of-change models, additional stages such as ‘*preparation*’ (i.e., stage in between contemplation and action where people are making preparation to take action by seeking information) and ‘*relapse*’ (i.e., failure to comply with the change made and return to old habit) have also been included [22, 23]. Overall, the stages-of-change model suggests that the individuals in later stages are most likely to help-seek, take up intervention, adhere to the intervention, and possibly to display successful outcome [20].

A study by Laplante-Lévesque et al. investigated the application of the stages-of-change model in audiologic rehabilitation in a sample of 153 adults with acquired hearing impairment seeking help for the first time [17]. They used the generic 24-item University of Rhode Island Change Assessment (URICA) scale as a measure of stages-of-change and identified four stages (i.e., precontemplation, contemplation, preparation and action) [24, 25]. According to URICA, 80% of the participants were in the action stage, 10% of participants in the contemplation stage, 8% were in the preparation stage and only 2% were in the precontemplation stage. Cluster analysis identified four stages-of-change clusters, which were named as: *active change* (58% of sample), *initiation* (35% of sample), *disengagement* (4% of sample), and *ambivalence* (3% of sample). Moreover, they found that the URICA scale had good construct validity, together with concurrent and predictive validity. Based on their observation, they suggested that change might be better represented on a continuum rather than movement in discrete steps. These observations are consistent with our previous studies on the “client journey” of individuals with hearing impairment where experiences were reported in multiple phases of the model [26–28]. This indicates that it might not always be possible to discretely categorize individuals exclusively to a single stage. Furthermore, the preparation stage was found to have the best concurrent and predictive validity, and this was identified as an area for future research [17].

In another recent study, Laplante-Lévesque et al. examined the stages of change in a sample of 224 adults who failed an online hearing screening [18]. According to URICA, 50% of the participants were in the preparation stage, 38% of participants in the contemplation stage, 9% were in the precontemplation stage and only 4% were in the action stage. Cluster analysis identified four stages-of-change clusters, which were named as: *decision making* (44% of sample), *participation* (28% of sample), *indecision* (16% of sample), and *reluctance* (12% of sample). Participants who reported a more advanced stage of change had significantly greater self-reported hearing disability, although they did not have a significantly worse speech-in-noise recognition threshold or significantly longer duration of hearing impairment.

With the stages-of-change model, it can be predicted that most individuals with hearing disability who see a clinician for help will be in the action stage and those who may be undergoing hearing screening may be in preparation stage [17, 18]. Assuming that this model also has good predictive validity with people from the general population, it is reasonable to assume that the rest of the population with hearing disability who are not seeking hearing-help actively may be in precontemplation, contemplation or preparation stages. If they are not aware of their hearing difficulties and/or in denial, they are likely to be in the precontemplation stage. However, if they are aware of their difficulties but not actively seeking hearing-help, then they are likely to be in contemplation or preparation stages. This assumption was supported by another study where 72% of older adults attending hearing screening were in precontemplation and contemplation stage [13]. However, they did not use the full URICA scale and instead used four questions (one for each stage) derived from the URICA. Participants had to choose one stage indicating their readiness to change. This may raise concerns about the construct validity of the questionnaire used in that study. For this reason the results must be viewed with caution and the differences in these studies make it difficult to compare them directly.

### Hearing disability and hearing help-seeking

It has been reported that self-reported hearing disability is more common than confirmed hearing impairment as measured using pure-tone audiometric testing (i.e., more people perceive and report hearing difficulties than are identified purely by pure tone audiometric threshold testing) [29, 30], making self-perceived hearing disability an important construct to measure. Indeed, a recent literature review suggested that self-reported hearing disability is one of the strongest predictors of hearing help-seeking, hearing aid uptake, hearing aid use, and satisfaction with hearing rehabilitation [31]. In addition, several studies suggest a significant relationship between hearing disability and health related quality of life when compared to measured hearing impairment [32].

Help-seeking and hearing-aid uptake among persons who are noticing hearing difficulties and persons with a confirmed hearing impairment, has received significant interest recently [31, 33–35]. Hearing aids are one of the main management options offered to individuals with hearing impairment. It is generally well known that hearing-aid uptake among those with hearing disability and hearing impairment is fairly low, and in developed countries it is estimated to range between 20 to 40% [36–38]. Most of the studies related to hearing loss, hearing help-seeking and hearing-aid uptake, has focused on those people who come to see hearing healthcare professionals in the clinic or attend a hearing screening. Little is known about characteristics of those with hearing disability in the general population who are noticing hearing difficulties but not seeking help, nor about those people who are aware of their hearing difficulties but do not want to adapt rehabilitation.

Further evidence is required for the application of stages-of-change model in rehabilitation audiology, with particular emphasis on understanding characteristics of those who are noticing hearing difficulties in the general population but not using interventions. The stages-of-change model could help identify the profiles and needs of these individuals, which may be valuable while developing appropriate interventions (e.g., pre-intervention counselling) to promote hearing help seeking and adoption of rehabilitation interventions in this population.

The aim of the current study was to test the hypothesis that adults experiencing hearing difficulties and aware of their difficulties but have not taken any action would fall under *contemplation* and *preparation* stages based on the transtheoretical stages-of-change model. In this study, we include both pre-clinical (i.e., those who are not seeking help) and clinical (i.e., those who are seeking help but not taking up rehabilitation) populations.

## Materials and Method

### Ethical considerations

Ethical clearance was obtained from Research Ethics Committee, College of Human and Health Sciences, Swansea University.

### Study design and participants

The study had a cross-sectional design, with data obtained through an online survey during a clinical trial (i.e., pre-intervention data) of a pre-fitting counseling program (Trial registration: ClinicalTrials.gov Protocol Registration System NCT01611129) [39, 40]. Clinical trial results have been presented elsewhere [40], however, here we report additional analysis using a different theoretical framework (i.e., TTM) that was not reported in earlier publications.

An advertisement was distributed in the United Kingdom though national newspapers, hearing loss charity websites (i.e., Action on Hearing Loss and Hearing Link) and local General Practitioner practice notice boards requesting “*those who were noticing hearing difficulties but not using hearing aids*” to participate in the study. All participants who completed the initial questionnaires (through an online survey) were included in the current study, however, only those who met the desired entry criteria, with higher self-reported hearing disability, were recruited for the pre-fitting counseling program [39, 40]. Those who were interested in participating in the study were directed to a website in which they were provided with information about the study and asked to complete the informed consent form and study questionnaires. A total of 90 participants completed the questionnaires and also provided demographic information. With this recruitment method we were able to reach people who were noticing hearing difficulties but not seeking help, and those who were aware of their hearing difficulties who consulted hearing healthcare professionals but were not taking up rehabilitation options. However, this excludes those who are not aware of their hearing difficulties and/or who are in denial.

### Measures

Stages of change, self-reported hearing disability, self-reported anxiety and depression and self-reported hearing disability acceptance were assessed with questionnaires administered via the Internet. Although the study aim was to investigate the stages-of-change using the URICA measure, additional measures were used to explore its association with other important constructs.

*Stages-of-change* were assessed using the URICA scale [21], which is the most commonly used stages-of-change measure that can be applied to most populations. The original URICA scale has 32 items (four stages with 8-items corresponding to each stage). Each item is rated on a 5-point Likert scale (1 = strong disagreement, 5 = strong agreement) and the total scores for each stage (i.e., subscale) can range from 8 to 40. In populations where the ‘maintenance’ stage is not appropriate a 24-item URICA has been used, which has three stages with 8-items corresponding to each stage (i.e., precontemplation, contemplation and action) [24, 25]. The generic URICA scale was used in this study by replacing the phrase ‘the problem’ with ‘the hearing problem’ to make it suitable for the current population. The same scale was used by Laplante-Lévesque et al. [17, 18] who identified four stages (i.e., precontemplation, contemplation, preparation and action). However, in the four stage model the precontemplation and action stages had 8-items each and contemplation and preparation had 3-items and 5-items respectively. In order to be able to compare the scores in each stage, weighted means were calculated for the contemplation and preparation stages as they had fewer numbers of items. The URICA scores were analysed and presented in four ways:

- *Stage scores*: These scores are used as a measure of stage endorsement and respondents can score high on more than one stage of change [41, 42].
- *Composite scores*: Two composites can be obtained using the different stage scores [43–46]. *Readiness to change composite* can be obtained by adding the scores of contemplation and action stage and subtracting the precontemplation score. *Committed action composite* can be obtained by subtracting contemplation stage scores from action stage scores. The higher the scores in these composites, the further along the participants are assumed to be in the stages-of-change model.
- *Stage with the highest scores*: This can be used to describe a respondent’s stage of change [23, 25]. With this, the respondent can only be in one stage at any point in time. For this reason, if two stages have equal scores then the stage furthest from precontemplation is considered to have the highest score.
- *Stages-of-change clusters*: Cluster analysis of URICA scores can produce stages-of-change clusters [21, 47]. Cluster analysis is a statistical technique which is used to group participants with similarity in their results. The sub-groups generated can help in better understanding the characteristics of the study population.

*Self-reported hearing disability* was assessed using the Hearing Handicap Questionnaire (HHQ) [48, 49]. The HHQ has 12-items with two subscales (emotional and social). Each item is rated on a 5-point Likert scale (1 = never, 5 = almost always) and the total scores in each subscale can range from 6 to 30 with higher scores indicative of more self-reported hearing disability (i.e., activity limitations and participation restrictions), and has been found to have good internal consistency, Cronbach’s alpha of .95 for emotional and .93 for social subscales [49].

*Self-reported anxiety and depression* symptoms were assessed using the Hospital Anxiety and Depression Scale (HADS) [50]. The HADS has 14 items with two subscales (anxiety and depression). Each item is rated on a 4-point Likert scale (0 = not at all, 3 = most of the time), and the total scores in each subscale can range from 0 to 21 with higher scores indicative of more self-reported anxiety and depression in respondents. The HADS has been found to have good internal consistency, Cronbach’s alpha of .80 for anxiety and .81 for depression subscales [51].

*Self-reported hearing disability acceptance* was assessed using the Hearing Disability Acceptance Questionnaire (HDAQ) [52], which was developed based on the Tinnitus Acceptance Questionnaire (TAQ) [53]. The HDAQ has 7-items with two subscales, which include: (1) activity engagement; and (2) avoidance and suppression. The subscale ‘activity engagement’ has 4-items and the subscale ‘avoidance and suppression’ has 3-items. Each item is rated on a 7-point Likert scale (1 = never true, 7 = always true) with items in subscale ‘avoidance and suppression’ having reverse scoring structure. The HDAQ was found to have a two-factor structure, which explains 75.69% of the total variance. In addition, the HDAQ has been found to have a good construct validity, concurrent validity (in relation to self-reported hearing disability, self-reported anxiety and depression and readiness to change measures), and relatively high internal consistency with Cronbach’s alpha of .89 for activity engagement and .82 for avoidance and suppression subscales [52].

### Data analysis

Statistical analysis was performed using the software IBM—SPSS Version 19 for Windows. In the first stage, descriptive statistics were used to examine demographic factors. Assumptions of normality and homogeneity of variance were tested and an alpha level of 0.05 was determined significant for all the statistical analysis. However, Bonferroni adjusted significance levels were used for interpretation of results to account for multiple comparisions (e.g., *p*<0.01 for comparison of sub-groups in stages-of-change clusters and analysis according to stage with highest scores). Various statistical tests were performed which include: (1) t-test to compare means between groups; (2) Pearson’s correlation coefficient to study correlation between composite scores and other factors; and (3) Hierarchical cluster analysis using Ward’s method [54], and standardised Z-scores to investigate the stages-of-change clusters. Evaluation of the cluster results was done with visual inspection of the dendogram (i.e., a tree diagram which shows a hierarchy of categories based on the degree of similarity or number of shared characteristics).

## Results

Table 1 shows the sample characteristics in the current study. As there is no clear consensus on the best method of reporting the URICA scores, we have reported results using all four different methods.

**Table 1:** Sample characteristics.

**Age (yrs; M ± SD)**		63.41 ± 10.49
**Gender (% female)**		50
**Years since hearing disability onset (yrs; M ± SD)**		
**Education (%)**	Compulsory education	13.3
	Secondary education	48.9
	Tertiary education	37.8
**Consulted hearing healthcare professional (%)**	Yes	65.6
	No	34.4
**Computer experience**	Basic	35.6
	Intermediate	61.1
	Expert	3.3
**Change assessment (URICA): Stages-of-change (Scores ± SD)**	Precontemplation	18.41 ± 3.30
	Contemplation	31.55 ± 4.17
	Preparation	30.66 ± 4.24
	Action	26.80 ± 5.30
**Change assessment (URICA): Composites (Scores ± SD)**	Readiness to Change	39.41 ± 8.63
	Committed Action	-4.20 ± 4.77
**Change assessment (URICA): Participants with highest scores in each stage (%)**	Precontemplation	0
	Contemplation	45.6
	Preparation	47.8
	Action	6.7
**Self-reported hearing disability (HHQ; M ± SD)**	Full scale	34.96 ± 9.95
	Emotional sub-scale	20.61 ± 5.75
	Social sub-scale	14.32 ± 4.85
**Self-reported anxiety and depression (HADS; M ± SD)**	Full scale	14.77 ± 7.50
	Anxiety sub-scale	7.04 ± 4.43
	Depression sub-scale	7.70 ± 3.81
**Self-reported hearing disability acceptance (HDAQ; M ± SD)**	Full scale	36.88 ± 7.85
	Activity Engagement sub-scale	22.72 ± 4.36
	Avoidance and Suppression sub-scale	14.16 ± 4.65

### Analysis according to stage scores

Stages-of-change scores can be used as a measure of stage endorsement. As predicted, a high percentage of participants (over 90%) in this study were in contemplation and preparation stages with the highest scores. Also, the participants had higher mean scores in contemplation and preparation stages, whereas precontemplation stage obtained the lowest mean scores.

### Analysis according to composite scores

The readiness to change composite score was 39.41±8.63 and committed action composite score was -4.20±4.77 in the current study sample.

Pearson’s correlation coefficient was performed to study the correlation among URICA composites with other factors. The readiness to change composite showed a moderate positive statistically significant correlation with the committed action composite [*r*(90) = .33, *p* < 0.01] and a weak negative and statistically significant correlation with self-reported hearing disability acceptance [*r*(90) = -.27, *p* < 0.01]. Although statistically significant correlation was observed, *R*-values are small explaining only 7% and 11% of the variability respectively in relation to committed action composite and hearing disability acceptance. For this reason, the relation between URICA score, committed action composite scores, and hearing disability acceptance measure are not very strong. However, no statistically significant correlation was observed between the readiness to change composite and other factors such as; duration of hearing disability, self-reported hearing disability and self-reported anxiety and depression. Also, no statistically significant correlation was observed between committed action composite and other factors such as; duration of hearing disability, self-reported hearing disability, self-reported anxiety and depression and self-reported hearing disability acceptance.

### Analysis according to the stage with the highest scores

Table 2 shows scores for various factors according to the stage with the highest scores. The precontemplation stage was excluded from analysis as no participants in this study were found to have a highest score in this stage. No statistically significant differences were observed among groups of stage with highest scores and factors such as: years since hearing disability, self-reported hearing disability, self-reported anxiety and depression, and self-reported hearing disability acceptance. Even though the scores for committed action composite scores increased with the stage with the highest score from contemplation to action, no statistically significant differences were observed among different groups based on the stage with the highest scores. Participants with the highest scores in the action stage had higher readiness to change composite scores when compared to participants with the highest score in the preparation stage [*t*(47) = -2.17, *p* < 0.05]. However, this effect was not found to be statistically significant considering the Bonferroni adjusted significance levels (i.e., *p*<0.01). Also, we found no statistically significant difference in scores among those participants with highest scores in the preparation stage and the action stage in terms of readiness to change composite.

**Table 2:** Years since hearing disability, self-reported hearing disability, self-reported anxiety and depression, self-reported hearing disability acceptance, readiness to change composite and committed action composite according to stage with highest scores (Note: Precontemplation was not in the analysis as n = 0 in this stage).

	Contemplation	Preparation	Action
**N**	41	43	6
**Years since hearing disability (M ± SD)**	11.49±8.37	12.07±12.97	10.00±10.52
**Self-reported hearing disability (HHQ; M ± SD)**	35.46±11.38	34.91±8.85	31.83±7.41
**Self-reported anxiety and depression (HADS; M ± SD)**	15.63±7.09	13.65±7.35	16.83±11.12
**Self-reported hearing disability acceptance (HDAQ; M ± SD)**	35.80±8.14	37.84±7.61	37.33±7.94
**Readiness to change composite (Scores ± SD)**	40.27±8.80	37.74±8.51	45.50±4.88
**Committed action composite (Scores ± SD)**	-4.49±18.10	-4.09±5.47	-3.00±2.53

### Analysis of stages-of-change clusters

**Fig 1:**
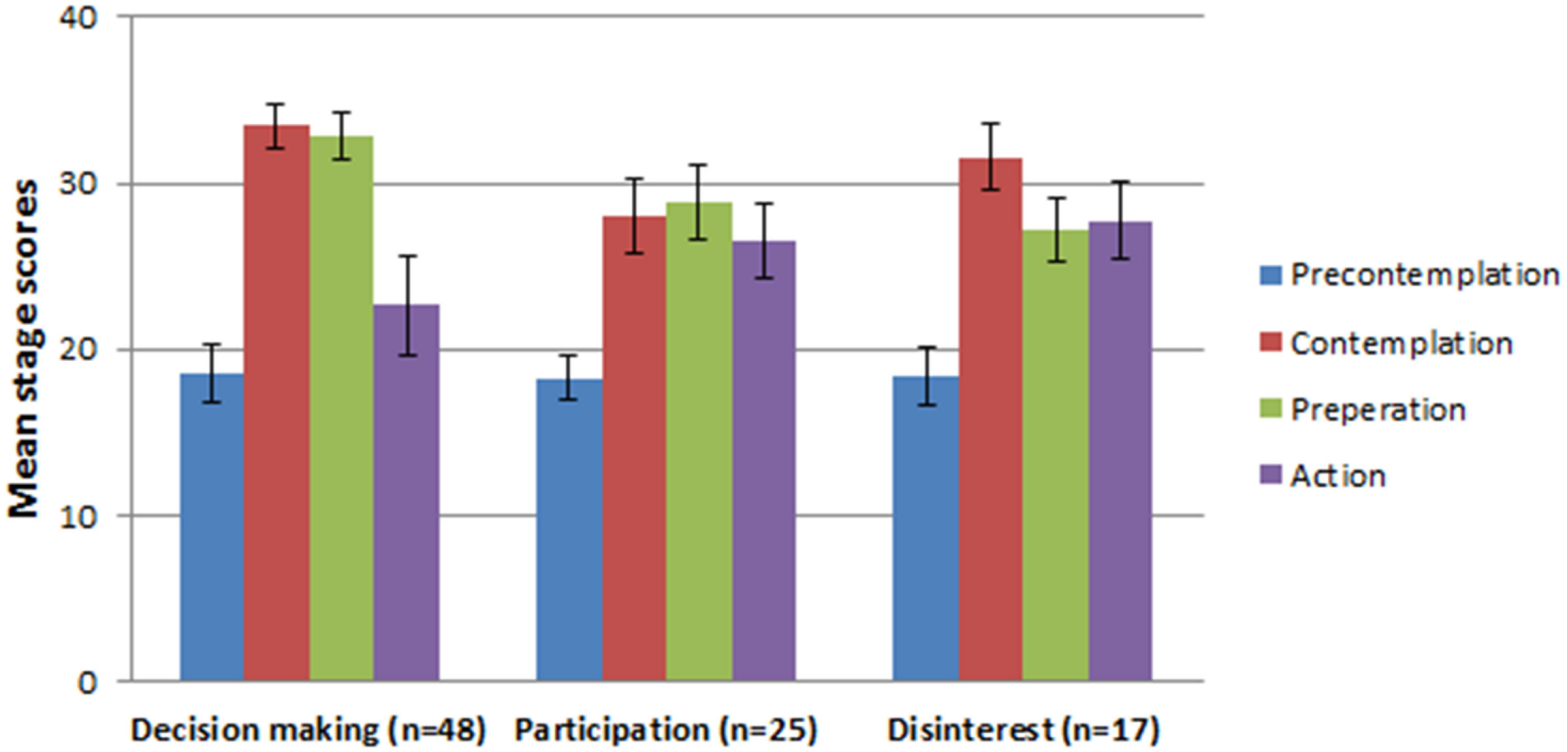
Stages-of-change clusters according to cluster analysis. **(**Error bars indicate standard deviation).

Hierarchical cluster analysis was performed using Ward’s method and standardised Z scores to identify the stages-of-change clusters in the sample participants. Cluster analysis can identify individuals who share similar characteristics in the same group when compared to other groups (i.e., sub-grouping individuals in a sample based on a set of criteria). Inspection of the dendogram identified three unique clusters. Fig 1 shows the mean stage scores for each of the clusters. Based on the profiles they represent, they were named as: (1) *decision making*; (2) *participation*; and (3) *disinterest*. Scores of the precontemplation stage did not vary among the three groups. However, differences in other stage scores (i.e., contemplation, preparation and action) made the groups distinct.

*Decision making* was the most common cluster, which represents 53% of the study population in which participants had equally high scores in contemplation and preparation stages when compared to action stage. The difference in the stage scores between contemplation and preparation were not significant. But the stage scores between contemplation and action stages *t*(47) = 7.18, *p* < 0.001, and also between preparation and action stages *t*(47) = 7.18, *p* < 0.001, were found to be statistically different. The *participation* cluster represents 28% of the study population in which the scores in all three stages (i.e., contemplation, preparation and action) were almost equal and no statistically significant difference were found between mean scores. Lastly, the *disinterest* cluster represents 19% of the study population in which participants had equal scores on preparation and action stage but higher scores in contemplation stage. The stage scores between contemplation and preparation *t*(16) = 3.87, *p* < 0.01, were found to be significantly different but not for scores between preparation and action stages. In addition, although some differences were noticed among scores between contemplation and action [*t*(16) = 2.79, *p* < 0.05], they were found not to be significant considering the Bonferroni adjusted significance levels (i.e., *p*<0.01).

## Discussion

The study was aimed at testing the hypothesis that adults experiencing hearing difficulties who have not taken any action would fall under *contemplation* and *preparation* stages based on the transtheoretical stages-of-change model. In addition, this study presents results of a stages-of-change measure and relates this measure with additional factors of hearing disability and acceptance, as well as possible related factors of anxiety and depression among adults noticing hearing difficulties who have not taken any action.

In the current study, most participants had their highest URICA scores in the contemplation or preparation stages as predicted by the stages-of-change model. These results are similar to recent study results of adults who failed online screening where 88% of participants had their highest URICA scores in contemplation and preparation stages [18]. This suggests some similarities between the current study sample and the previous study sample of those who failed an online hearing screening. If the participants had been in denial, they may have been in the precontemplation stage. However, the fact that they were aware of difficulties and started to make efforts to seek information, and also as shown by volunteering to participate in this study, suggest that they were in contemplation or preparation stages. It is also important to note that 65% of participants had consulted health professionals about their hearing difficulties, which suggests that the participants are aware of their difficulties; hence they are not in the precontemplation stage. However, we do not have further information about the consultation (e.g., if they had a hearing test or the information provided by hearing healthcare professionals), nor documented reasons for not taking up interventions. As mentioned earlier, previous studies in relation to hearing impairment have identified most participants as being in precontemplation or in action stages, depending on the study population [13, 17].

There were some differences observed among URICA composite scores (i.e., readiness to change and committed action) between the current study sample and those with hearing impairment seeking help for the first time [17], but not with those who failed online hearing screening [18]. However, the study recruitment strategy (i.e., advertisement via national newspaper, charity websites, etc) may have attracted a mixture of participants from the general population (i.e., pre-clinical population which includes those who are not actively seeking help; and the clinical population who consulted hearing healthcare professionals for further help but decided not to take up rehabilitation at that point in time). This may have had some influence on the study results as the previous study by Laplante-Lévesque et al. [17] was focused on the clinical population with confirmed hearing impairment. Also, statistically significant positive correlations were seen between readiness to change and committed action composites in the current study. However, statistically significant negative correlations were observed between readiness to change composite and hearing disability acceptance. This is possibly because the HDAQ measures acceptance more in line with psychological acceptance (i.e., recognising a condition without attempting to change it) rather than in help-seeking and hearing aid uptake [52].

There was no linear relationship found between other factors such as duration of hearing disability, self-reported hearing disability, self-reported anxiety and depression, and self-reported hearing disability acceptance among groups based on stage with highest scores. This may be to some degree related to not having any participants in precontemplation stage and having very few participants in the action stage. However, these findings may raise some concerns about the concurrent validity of the stages-of-change measure. Further studies are necessary to evaluate if other factors have linear relationship with stages-of-change as indicated in the previous study [17].

Cluster analysis identified three distinct stages-of-change clusters (see Fig 1), which were very different from the clusters reported by Laplante-Lévesque et al. [17], which included: *active change*, *initiation*, *disengagement* and *ambivalence*. However, two clusters (i.e., *decision making* and *participation*) had the same characteristics as reported in a recent study on adults who failed online hearing screening and hence we used the same names for them [18]. 81% and 72% of participants were in these two clusters in the current study and the previous study [18] respectively. Some similarities and differences can be noticed when comparing these results to clusters reported with adults engaged in psychotherapy and in people with arthritis [21, 47]. Most participants in the current study were in the *decision making* cluster (equal but significantly higher scores in contemplation and preparation stages when compared to action stage). Hence, they are still probably not ready to make an action but seeking information. Approximately one fourth of the population were in the *participation* cluster (equal scores in contemplation, preparation and action stages with no statistically significant difference among these scores) who would probably be close to taking action. The rest of the participants were in the *disinterest* cluster (significantly higher scores in contemplation stage scores when compared to preparation and action stage scores). People in this group may be thinking about the problem but not seeking information actively, and this group may take longer to make a change. However, due to the cross-sectional design of the study, no firm conclusions can be drawn about the longitudinal predictive validity of these findings.

It has been highlighted in previous studies that the staging algorithms might be based on arbitrary time periods and may not be measuring discrete stages of change [55]. The current study results support the arguments that the change might be better represented as a continuum (i.e., stages-of-change scores and the cluster analysis results) rather than discrete stages [17]. However, the discrete stages might be pedagogically useful to understand the change in a simple way, and possibly for counselling purposes as suggested in studies on patient journey [26–28].

### Implications of the study

This study could have a number of applications. First, this information about stages of change characteristics could be useful information for various stakeholders such as government, hearing aid industry, hearing-related charities that could use this information while planning hearing health—awareness events, and promotion and education by developing targeted approaches to different groups of population, if these results are replicable in further studies. Second, it is also common that some people with hearing difficulties who are not motivated to seek help come to see hearing healthcare professionals due to various other reasons (e.g., persuasion from communication partners). For this reason, it is important to better understand the characteristics of such a population. Furthermore, it is suggested in the literature that adults with hearing impairment take about 10 years on an average to seek help after they have started to notice hearing difficulties [56]. However, an international online survey suggests that the number of years taken for those to help seek after they started noticing hearing difficulties does not have a normal distribution [57]. Rather, the study identified clusters in the population showing a bimodal distribution, with some seeking help in the first few years (i.e., 1–3 years) and others taking action for the first time after 10 years or more. This makes the average duration approximately 7 years. Although the survey results have to be viewed with caution as they are not published in a peer reviewed journal, these new findings suggest that there are different groups of people and it is important to identify those who are in the earlier stages (i.e., precontemplation, contemplation and preparation) and provide them with necessary information which may help them to make appropriate decisions about hearing help-seeking.

### Limitations of the study and future research

The study has some limitations that were unavoidable, mainly due to the nature of study sample chosen. The data was collected using self-reported measures using the internet. This method of data collection may have eliminated some people who do not have access to internet and also those who cannot use internet due to other disabilities (e.g., visual impairment) and may not be representative of the general population [58, 59]. Even though this is a limitation, it is very challenging to reach this particular population who do not come to see clinicians, and also those who consult with clinicians but do not take up recommended interventions. For this reason, and also to obtain data from across the country, this method was regarded as appropriate. Also, results obtained from an online format of questionnaires completion may not be identical to the pen-and-paper format [60], even if measurement characteristics tend to be the same or even better for internet administration [61]. In this study, the researchers did not make a direct contact with participants. However, we were unable to find any studies in the field of audiology which investigated whether individuals who participate in a study without researchers contact respond in the same way to those who do have contact with researchers.

Comparison of our results with the previous study by Laplante-Lévesque et al. [17] may reveal some additional limitations. This is because the sample in the previous study included only those with confirmed hearing impairment, which may have excluded a small percentage of those who are noticing hearing difficulties but found to have no measurable hearing impairment. However, in the current study we had a population with self-reported hearing disability but no confirmed hearing impairment. There are discrepancies between self-reported hearing disability and hearing impairment, and it is reported that self-reported hearing disability is more frequent than hearing impairment as defined by audiometric results [29, 62]. This means the current study probably had a broader population than the previous study in terms of hearing characteristics [17], but a major limitation is the lack of audiometric data or diagnostic speech tests that would confirm the presence or absence of hearing loss. Moreover, although we targeted those who are noticing hearing difficulties in the general population, it is likely that those who are considering seeking help may be more likely to have responded to the questionnaires. This automatically excludes those who are in denial, resulting in some recruitment bias in this sample.

The current study was limited in terms of the main factors included (i.e., stages-of-change, duration of hearing disability, self-reported hearing disability, self-reported anxiety and depression and self-reported hearing disability acceptance). Other factors such as attitude and motivation could have also been useful in better understanding the population characteristics. Although the cross-sectional design helped us understand the population characteristics, no predictions can be made about their help-seeking behaviour from these findings and longitudinal studies are necessary with particular emphasis to process of change. Furthermore, even though Milstein and Weinstein have reported some findings in the population who are noticing hearing difficulties but in denial (i.e., those in precontemplation stage), not much is known about them and further studies are necessary to better understand characteristics of that population [13]. Moreover, as the communication partners (CPs) play an important role in persuading people with hearing disability to seek help and adapt intervention [63], it would also be useful to study the characteristics of CPs in addition to person with hearing disability.

## Conclusions

The study was aimed at testing the hypothesis that adults experiencing hearing difficulties who have not taken any action would fall under *contemplation* and *preparation* stages based on the transtheoretical stages-of-change model. The study sample included both pre-clinical (i.e., those who are not seeking help) and clinical (i.e., those who are seeking help but not taking up rehabilitation) populations, but not those who were in denial. The majority of the participants (over 90%) in this study were in contemplation and preparation stage as predicted from the model. At a population level, the stages-of-change model can predict overall readiness to change and also the stage at which the people with hearing disability may be categorized. The current study results support the arguments that the change might be better represented as continuum rather than discrete stages (e.g., precontemplation, contemplation, preparation and action). The potential applications of stages-of-change model in relation to hearing disability and its application to audiological rehabilitation needs to be further investigated.

## Supporting Information

S1 Dataset (XLSX)
